# MuscleJ: a high-content analysis method to study skeletal muscle with a new Fiji tool

**DOI:** 10.1186/s13395-018-0171-0

**Published:** 2018-08-06

**Authors:** Alicia Mayeuf-Louchart, David Hardy, Quentin Thorel, Pascal Roux, Lorna Gueniot, David Briand, Aurélien Mazeraud, Adrien Bouglé, Spencer L. Shorte, Bart Staels, Fabrice Chrétien, Hélène Duez, Anne Danckaert

**Affiliations:** 1 Inserm, CHU Lille, Institut Pasteur de Lille, University of Lille, U1011 - EGID, 1 rue du Pr. Calmette, F-59000 Lille, France; 2 Experimental Neuropathology Unit, Infection and Epidemiology Department, Institut Pasteur, 25, rue du Docteur Roux, 75015 Paris, France; 3 UTechS PBI (Imagopole)–Citech, Institut Pasteur, 25, rue du Docteur Roux, 75015 Paris, France

**Keywords:** Skeletal muscle fiber, Histology, Image automated quantification, In situ cartography, Fiber typing, Satellite cells, Stem cells, Vessels

## Abstract

**Background:**

Skeletal muscle has the capacity to adapt to environmental changes and regenerate upon injury. To study these processes, most experimental methods use quantification of parameters obtained from images of immunostained skeletal muscle. Muscle cross-sectional area, fiber typing, localization of nuclei within the muscle fiber, the number of vessels, and fiber-associated stem cells are used to assess muscle physiology. Manual quantification of these parameters is time consuming and only poorly reproducible. While current state-of-the-art software tools are unable to analyze all these parameters simultaneously, we have developed MuscleJ, a new bioinformatics tool to do so.

**Methods:**

Running on the popular open source Fiji software platform, MuscleJ simultaneously analyzes parameters from immunofluorescent staining, imaged by different acquisition systems in a completely automated manner.

**Results:**

After segmentation of muscle fibers, up to three other channels can be analyzed simultaneously. Dialog boxes make MuscleJ easy-to-use for biologists. In addition, we have implemented color in situ cartographies of results, allowing the user to directly visualize results on reconstituted muscle sections.

**Conclusion:**

We report here that MuscleJ results were comparable to manual observations made by five experts. MuscleJ markedly enhances statistical analysis by allowing reliable comparison of skeletal muscle physiology-pathology results obtained from different laboratories using different acquisition systems. Providing fast robust multi-parameter analyses of skeletal muscle physiology-pathology, MuscleJ is available as a free tool for the skeletal muscle community.

**Electronic supplementary material:**

The online version of this article (10.1186/s13395-018-0171-0) contains supplementary material, which is available to authorized users.

## Background

The plasticity of skeletal muscle refers to its ability to adapt to environmental changes and its potential for regeneration. During embryonic development, Pax7-positive muscle progenitor cells enter the myogenic program by activating the expression of myogenic regulatory factors (MRF) and give rise to primary myofibers [1]. During fetal and post-natal periods, proliferative Pax7-positive cells contribute to skeletal muscle growth while some of them adopt quiescence. These adult muscle stem cells are named satellite cells due to their peripheral position under the basal lamina and the sarcolemma of muscle fiber. They are required for skeletal muscle regeneration after injury [2]. Myofibers express different myosin isoforms that are related to specific properties of ATP hydrolysis and, therefore, muscle fiber contraction. Two classes of muscle fibers can be distinguished: slow-type oxidative fibers, which are more resistant to fatigue, consume more oxygen, and express type I myosin; and fast-type glycolytic fibers, which generate more force, express type II myosin (type IIA, IIX, IIB). During adult life, external signals, e.g., hormones or exercise, can switch the fiber type initially established during development and post-natal development [3]. Environmental changes can also affect skeletal muscle mass by adapting myofiber diameters, which can result in perturbations of skeletal muscle function. This is observed, for example, in aging-induced atrophy or in steroid-induced hypertrophy [4]. Understanding the mechanisms underlying skeletal muscle plasticity is essential for the development of novel therapies targeting skeletal muscle dystrophies, sarcopenia, cachexia, and metabolic disorders.

Strategies to dissect the molecular mechanisms of such processes are usually based on the invalidation or overexpression of specific genes, modulation of metabolism, or activation of pharmacological targets in different animal models. Because different cell types are present in skeletal muscle, most of these experiments use histological techniques to specifically analyze the morphology of myofibers. Fiber typing, which aims to establish the proportion of each fiber type (I, IIA, IIX, IIB), is often performed in studies related to skeletal muscle metabolism. Atrophy and hypertrophy are assessed by measuring skeletal muscle cross-sectional area (CSA). Concerning skeletal muscle regeneration, one important characteristic is the presence of nuclei located in the center of fibers (centronuclei), resulting from the fusion of newly formed myoblasts with each other or with existing myofibers [5]. This is distinguished from undamaged fibers, which, in mice, display peripheral nuclei. Consequently, the number of centronuclei per fiber and CSA of regenerated fibers are readouts of skeletal muscle regeneration efficiency. In addition, the number of fiber-associated satellite cells informs on their potential to perform successive rounds of degeneration/regeneration. These experiments and analyses are time consuming, and different semi-automatic and automatic tools have been developed over the years based on different software and strategies [6–10]. Among them, TREAT-NMD SOP DMD_M.1.2.001 (2008) is an automated image analysis method running on the commercial CellR Software (Olympus) that allows determination of fiber diameter and detection of centronuclei in segmented fibers. Another application, Semi-automatic Muscle Analysis using Segmentation of Histology (SMASH), is developed in the MATLAB environment. It provides fiber properties and typing, centronucleated fiber detection as well as quantification of inter-fiber objects (e.g., capillaries), after semi-automatic fiber segmentation on immunofluorescent images, which requires manual adjustments [8]. Finally, the recently developed open source MyoVision software provides the number of fibers per section, CSA, minimum Feret diameter, fiber type, and number of myonuclei [7]. However, none of these methods are designed to provide either the number of nuclei in centronucleated fibers, the CSA of different types of fibers (e.g., centronucleated fibers vs normal fibers or type I fibers vs type IIA fibers), nor the number of fiber-associated satellite cells. In addition, the diversity of the available software makes it time-consuming to master each of them for studying different parameters in one study. Consequently, while many tools have been developed to automate histological analysis of skeletal muscle, most quantification is still performed manually using the well-known and open source Fiji (Fiji is just ImageJ) platform [11]. In this study, we present a new, complete high-content skeletal muscle analysis tool developed on this software that we named MuscleJ. It presents a number of advantages over existing methods as it groups most parameters frequently studied in fluorescence by the skeletal muscle community.

The tool we developed uses images coming from acquisition of immunofluorescent staining. It can perform segmentation of skeletal muscle fibers and quantification of centronucleated fibers and the number of centronuclei per fiber. It calculates fiber CSA and discriminates the CSA of regenerated centronucleated fibers from uninjured fibers. In addition, it quantifies myonuclei and fiber-associated Pax7-positive satellite cells and vessels. Finally, it also quantifies up to three different intramyofiber labelings, which could correspond to different myosins for the establishment of the fiber typing, or other parameters depending on the antibodies used or research question. It is completely automated, and the only requirement is working with high-quality images obtained by microscopy. In addition, we have implemented, for the first time, optional cartographies offering the possibility to get a rapid overview of the results on reconstituted skeletal muscle section images. This allows visualizing the number of vessels and satellite cells per fiber or the repartition of centronucleated fibers in the section, representing the extent of an injury. The cartography showing the fiber CSA is also useful to rapidly visualize skeletal muscle atrophy or hypertrophy. Furthermore, the speed of this complete automated method makes it possible to significantly increase the amount of fibers analyzed and, therefore, improve statistical power of the analyses.

## Methods

### Mice and tissue preparation

Seven- to twelve-week-old wild-type mice were used. Mice were euthanized by cervical dislocation, and skeletal muscles (tibialis anterior and gastrocnemius) were snap-frozen in liquid nitrogen-cooled isopentane and stored at − 80 °C for cryosectioning.

To induce skeletal muscle injury, mice were first anesthetized by intraperitoneal injection of ketamine (100 mg/kg)/xylazine (10 mg/kg) and 15 μl of notexin (12.5 μmol in 0.9% NaCl, Latoxan, France) was injected in the tibialis anterior.

### Immunofluorescent staining

Immunofluorescent staining was carried out on skeletal muscle (tibialis anterior and gastrocnemius) frozen sections (7 to 12 μm). For satellite cell staining, sections were incubated for 6 min in a methanol bath at − 20 °C followed by 10 min in a bath at 100 °C containing antigen retrieval buffer (Vector #3300) diluted 1:10 in PBS1X. Sections were blocked in PBS 1X- 3% BSA or 5% horse serum. Primary and secondary antibodies are described in Table 1.

**Table 1:** List of antibodies and conditions in which they were used

		Primary antibody	Secondary antibody
Fiber/nuclei (from 2 labs)	Laminin	Laminin α-2 (4H8-2) Santa Cruz #59854 (1/250) ON 4 °C	Donkey anti-rat IgG Alexa Fluor 488 ThermoFisher #A-21208 (1/500) 45 min RT
	Hoechst		Hoechst 33342 ThermoFisher #H3570 (1/1500) 45 min RT
	Laminin	Anti-laminin Ab (produced in Rabbit) L9393 thermoscientific (1/100) ON 4 °C	Pierce donkey anti-rabbit IgG (H+L) cross adsorbed secondary Ab DyLight 650 (prod#Sa5-1041) (1/200) 1 h RT
	Hoechst		Hoechst 33342 ThermoFisher #H3570 (1/1000) 1 h RT
Satellite cell	Laminin	Anti-laminin Ab (produced in rabbit) L9393 thermoscientific (1/100) ON 4 °C	Pierce donkey anti-rabbit IgG (H+L) cross adsorbed secondary Ab DyLight 650 (prod#Sa5-1041) (1/200) 1 h RT
	Pax7	Monoclonal mouse anti-Pax7(supernatant), Developmental Studies Hybridoma Bank (1/20) ON 4 °C	Biotinylated horse anti-mouse IgG (H + L) (Vector Laboratories, #BA-2000) = amplification (1/200) 1 h RT + Streptavidin-DTAF (Beckman Coulter, #PN IM0307) (1/1000) 1 h RT
Vessels	Laminin	Anti-laminin Ab (produced in rabbit) L9393 thermoscientific (1/100) ON 4 °C	Pierce donkey anti-rabbit IgG (H + L) cross adsorbed secondary Ab DyLight 488 (prod#Sa5-10038)
	CD31	BD Pharmigen 550,274 purified rat anti-mouse CD31 (1/50) ON 4 °C	Pierce donkey anti-rat IgG (H + L) cross adsorbed secondary Ab DyLight 550 (Prod#Sa5-10027) (1/200) 1 h RT
Fiber type	Type I	BA-D5 (mouse IgG2b) DSHB (1/100) 1 h 37 °C	Anti-mouse IgG2b 647 nm life technology A21242 (1/250) 30 min 37 °C
	Type IIA	SC-71 (mouse IgG1) DSHB (1/100) 1 h 37 °C	Anti-mouse IgG1 568 nm life technology A21124 (1/250) 30 min 37 °C
	Type IIB	BF-F3 (mouse IgM) DSHB (1/100) 1 h 37 °C	Anti-mouse IgM 488 nm life technology A21042 (1/250) 30 min 37 °C
	Laminin	anti-laminin (rabbit) DSHB(1/100) 1 h 37 °C	Anti-rabbit 4+A2:D1905nm abcam ab175651 (1/250) 30 min 37 °C

### Image acquisition

All images were acquired using the Axio Scan. Z1 (Zeiss). The acquisition settings are summarized in Table 2. However, other acquisition systems have been tested (Additional file 1: Table S1), such as an Apotome widefield (Apotome Axio Observer Z1, Zeiss), a confocal microscope (LSM 700 AxioObserver, Zeiss), and a spinning disk confocal (Cell Voyager CV1000, Yokogawa, Japan).

**Table 2:** Acquisition settings of tested images for the developed analysis feature

Acquisition		Analysis			
		centro-nucleated fibers	Vessels	Satellite cells	Fiber type
Image dimensions	Z-Stack	No	No	No	No
	Channels	2	3	3	4
	Scaling xy (per pixel)	0.325 μm × 0.325 μm	0.325 μm × 0.325 μm	0.38 μm × 0.38 μm	0.325 μm × 0.325 μm
Acquisition information	Microscope	AxioScan.Z1	AxioScan.Z1	LSM 700	AxioScan.Z1
	Objective	20×	20×	40×	20×
Channel 1	Reflector	DAPI	DAPI	DAPI	DAPI
	Excitation wavelength	353	353	404	353
	Emission wavelength	465	465	444	465
Channel2	Reflector	FITC	Cy3	Cy3	Cy3
	Excitation wavelength	495	548	561	548
	Emission wavelength	519	561	575	561
Channel 3	Reflector		FITC	FITC	FITC
	Excitation wavelength		495	488	495
	Emission wavelength		519	517	519
Channel 4	Reflector				Cy5
	Excitation wavelength				650
	Emission wavelength				673

### Image analysis

The automated image analysis workflow was implemented in Fiji (NIH, Bethesda, MD, USA) [11] environment as a macro. Analysis was performed on different Windows/Mac OS computers with the following minimum requirements:

- RAM: 16 GB
- System type: 64 bits operating system
- Fiji version: from 1.51e, presently tested on 1.51n
- Java version: Java 1.8.0–66 (64 bits)
- Used plugins: Bio-Formats plugins for Fiji (release 5.5.3)
- Main used Fiji functions: the main used functions accessible from internal libraries have been listed in Additional file 1: Table S2

### Method validation

Comparison between control and regenerated skeletal muscle, 3 weeks after myotoxin intramuscular injection, was used to validate the detection of centronucleated fibers by MuscleJ, as was first described in [5]. Five independent experts with strong experience in skeletal muscle biology from two independent labs received a random set of images. Using Fiji software, and following specific instructions, they manually quantified fiber size, number of centronucleated fibers, fiber typing, and number of vessels and satellite cells. These results were compared to those obtained from MuscleJ. A second validation was performed by two independent experts to manually track individual fibers and determine their CSA, as well as the number of nuclei, in each centronucleated fiber on Fiji.

### Statistics

Prism 6.0 (GraphPad Software Inc.©, USA) was used for statistical analysis. Data were analyzed by Mann–Whitney test or Student’s *t* test after being assessed for normality of sample distribution. Inter-condition sample variability was tested by Kruskal–Wallis one-way analysis of variance. Qualitative traits (i.e., fiber type distribution) were analyzed by a chi-square (*χ*^2^) test. Statistical significance is shown on the graphs (*p < 0.05; **p < 0.01; ***p < 0.001; ****p < 0.0001).

Concordance matrix between the experts and MuscleJ classes as fiber type or CNF classes have been processed to calculate classification accuracy by feature. Statistical tests used for each data set are indicated in the figure legends.

### Tutorial

A step by step tutorial is given in Additional file 1: Tutorial.

## Results

### Overview of the MuscleJ algorithm: multi systems and multi channels (Fig. 1a)

Different acquisition systems were used to obtain images from muscle sections stained with DAPI and a laminin antibody. Among them, the Apotome (Zeiss), confocal LSM700 (Zeiss), and spinning disk CV1000 (Yokogawa, Japan) can produce reconstituted images of entire muscle sections after generation of mosaics. However, acquisition with these systems is time consuming. Conversely, the AxioScanZ1, which was also used to generate widefield muscle images, offers the advantage of giving high quality images faster. Therefore, we used this system for the majority of this study to generate high-content data. However, MuscleJ is designed to analyze images coming from a variety of systems. Image pretreatment depends on the scanned surface, the number of z slices acquired, and the number of channels. Up to four channels can be treated simultaneously. MuscleJ adapts the process to these different parameters automatically, leading to better recognition of muscle fiber outlines and fluorescent signals of interest.

**Fig. 1:**
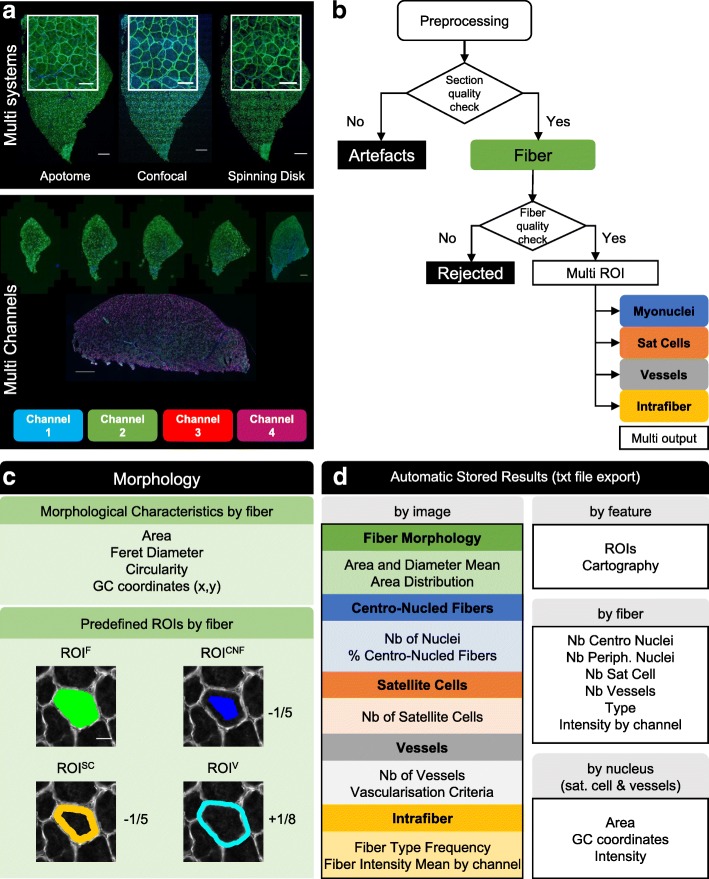
Overview of the MuscleJ workflow and feature pipeline. **a** Images of the multi-system panel represent the same muscle section acquired by different microscopes: Apotome (Zeiss-25X), Confocal LSM700 (Zeiss-25X), Spinning Disk CV1000 (Yokogawa-20X), and Scale Bar (SB) equals 400 μm. Part of the image appears at higher magnification in white outline, SB equals 200 μm. In the multi-channels panel, images were obtained from the AxioscanZ1 (Zeiss-20X) and muscle sections, with different staining are represented by slide and section. SB equal respectively to 500 μm. **b** Representation of the MuscleJ organigram. **c** Automatic detection of different region of interest (ROI) of skeletal muscle fibers, based on laminin staining (gray), corresponding to regions in which several parameters are analyzed (F fiber, CNF centronucleated fiber, SC satellite cell, V vessels). ROI^CNF^, ROI^SC^ and ROI^V^ are proportional to the minimum Feret diameter of fibers (− 1/5, − 1/5 and + 1/8 respectively). SB equals 20 μm. **d** List of the different outputs obtained from MuscleJ, per image, feature, fiber, or nucleus (Nb Number)

### MuscleJ organigram (Fig. 1b)

The first step of the program is preprocessing. In this stage, all muscle fibers of the section are segmented. A ratio comparing the area of the entire muscle section with the sum of all segmented fiber areas is automatically established during this step and serves to determine the quality of images. The percentage of accepted artefact is indicated by the user in the initial dialog box. MuscleJ then controls the decision to continue the analysis or exclude the image as an artifact, as illustrated in the Additional file 1: Figure S1A. This function, which works as a quality control, avoids the analysis of a partial or non-representative part of the section. After validation, fiber morphology is processed in the images. A second quality check occurs to eliminate poorly segmented fibers (associated with poor staining) that would otherwise represent bias in the analysis. For that, MuscleJ measures the CSA of all fibers of the section and eliminates those bigger than the average of fiber CSA plus three times the standard deviation. However, MuscleJ is adaptable to different physiological/phenotypical conditions. This was confirmed by an analysis performed on Mdx mice, a model presenting a wide range of fiber sizes. In this model, both small and large fibers are correctly quantified on the same muscle section, by MuscleJ (Additional file 1: Figure S2).

For segmented fibers, different regions of interest (ROI) are automatically saved and used for the analyses selected in the initial dialog box. Specific ROIs are created with the detection of myonuclei, satellite cells, vessels, or intramyofiber staining, and results are then automatically saved as tables and graphs in appropriate formats.

### Data analysis with MuscleJ (Fig. 1c, d)

Each muscle fiber is segmented by MuscleJ after a pretreatment consisting of background reduction and contrast enhancement. This leads to precise segmentation of the laminin signal, which stains the basal lamina of fibers. At this stage, different parameters are obtained, including the CSA, circularity, and the minimal and maximal Feret diameters as well as the position of each fiber within the section by gravity center (GC) determination. A mask of this ROI corresponding to the fiber and named ROI^F^ (region of interest of fiber) is created and saved (in green, Fig. 1c). It will be used for all other functions. Therefore, a high-quality laminin staining is essential for MuscleJ analysis. Three other ROIs are defined by MuscleJ. For detection of centronucleated fibers (CNF), we defined a mask corresponding to one fifth of the minimal Feret diameter for the creation of the ROI^CNF^ (region of interest of centronucleated fiber), which represents the region in which the presence of myonuclei are considered as central (Fig. 1c). The space existing between the ROI^F^ and ROI^CNF^, which we named ROI^SC^ (region of interest of satellite cell), corresponds to the space occupied by satellite cells and non-central myonuclei (Fig. 1c). Finally, we defined the ROI^V^ (region of interest of vessel) as the space outside of the fiber corresponding to one of eighth of the minimal Feret diameter of the ROI^F^ (Fig. 1c). This mode of calculation for the establishment of the different ROIs has the advantage to be proportional to the fibers and can be therefore used for any fiber size. With the four ROIs predefined by MuscleJ, a precise and complete phenotype can be assigned to each fiber.

For each feature requested by the user (Fig. 1d), a specific algorithm is applied and allows fine and reproducible analysis on high numbers of fibers. For centronucleated fiber detection, the first step is detection of nuclei in the DAPI channel, followed by their localization in the ROI^CNF^. Each fiber with nuclei in this region of interest is recorded as a centronucleated fiber, and the number of centronuclei in the ROI^CNF^ is also quantified and saved. Another output from this analysis is the number of peripheral myonuclei, i.e., nuclei in the ROI^SC^ located between the ROI^F^ and the ROI^CNF^. For the analysis of satellite cells, a first segmentation of Pax7-positive cells is made over the entire section as well as the nuclei segmentation in the DAPI channel. For each Pax7-positive cell, a new function called by MuscleJ checks if the overlap between Pax7-positive detected cells and nuclei is sufficient (90% minimum) to consider them Pax7/DAPI double-positive satellite cells. A final check is made to ensure that these Pax7/DAPI-positive cells are correctly positioned between the fiber and the basal lamina in the corresponding ROI^SC^. In the same way, the detection of vessels is first realized by detecting CD31-positive cells, followed by checking their position in the ROI^V^. Two vascularization criteria are also provided by MuscleJ. The first corresponds to the number of CD31-positive cells per square millimeter, and the second to the percentage of the section occupied by the vessels. Morphological characteristics of vessels and satellite cells (area, intensity and gravity center coordinates) are also saved in a separate file and can be used for further analysis. The minimum distance between satellite cells and vessels can thus be calculated.

Finally, the function allowing the detection of intrafiber staining, e.g., those detected for fiber typing, is based on analysis of the intensity histogram of the respective channels. A threshold resulting from the specific signal-to-noise ratio is generated and then applied to ROI^F^ in order to discriminate positive and negative fibers. Each specific threshold is indicated in the summary table of results at the end of the process.

During a batch run, a table for each analyzed section is created and saved in a readable file format (txt file format), thus storing fiber phenotype details (Fig. 1d). In parallel, the ROIs defined after morphometry analysis are saved and readable by ROI Manager (Fiji function). At the end of the process, a new table summarizes results by section, allowing a data analysis overview (Fig. 1d). All these results and ROIs are saved in the folder previously designated by the user including reconstructed cartographies, if selected. Thus, the ROIs may be reused for further analysis.

### MuscleJ feature dialog boxes (Fig. 2)

**Fig. 2:**
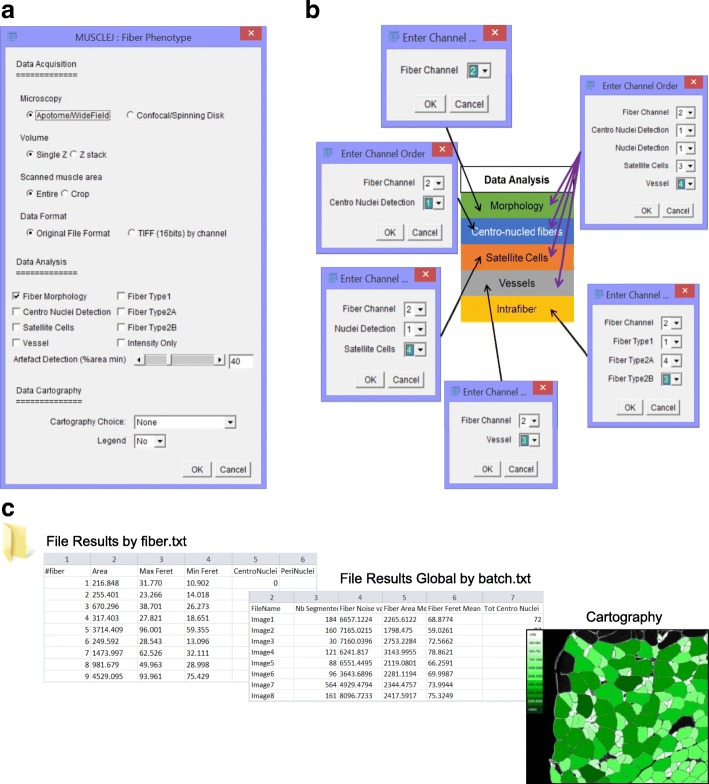
MuscleJ implementation. **a** Representation of the principal dialog box of MuscleJ with different sections: data acquisition settings, multi-data analysis choices, and in situ cartography representation. **b** Following option selection in **a**, a second dialog box appears and should be informed on the channels order in original file format. **c** Data are saved in global tables where the requested information is filled in, as well as details for each muscle fiber. Selected cartographies are also saved at this step

The first dialog box, named “MuscleJ Fiber Phenotype,” allows selection of different options (Fig. 2a). Concerning the “data acquisition,” users can choose the type of microscope used for image acquisition, select if a z-stack was performed, and the file format of images to be processed. Original format or TIF (16 bit) files can be used by MuscleJ. When the “z-stack” option is selected, an automatic Maximum Intensity Projection is performed by MuscleJ. The second part, related to “data analysis,” concerns the selection of different image parameters to be analyzed. Finally, the last section of the dialog box is the selection of “data cartographies” that can be performed on images with the option of specific legends directly on in situ cartographies.

When this first dialog box is accepted, a second one appears and requests information about channel order in the original file or in a series of individual channels for the TIF format, in correspondence with the options chosen in the previous dialog box (Fig. 2b and Additional file 1: Tutorial). After asking for the path where the images are to be read and results to be saved (Fig. 2c), the batch is executed for processing of a set of images, with the corresponding characteristics previously selected. Results are saved in the output folder that will contain four new folders: a folder named “Artefacts” containing images not accepted by the quality check, a folder named “Cartography” where Jpeg images of cartographies are saved, a folder named “Results by file” containing results of all the individual text files of each image of the batch (by fiber), and a folder named “ROI” where the ROI_CNF, ROI_F, ROI_SC, ROI_V for each fiber of each image of the batch are saved. These ROI can be applied in the future for further analyses. In addition, a text file named “RunGlobalResult_xx” is generated containing a summary of all the results of the batch based on the analysis performed (i.e., FM fiber morphology, SC satellite cell detection, V vessel detection).

### Implementation of in situ cartography to score skeletal muscle phenotypes (Fig. 3)

**Fig. 3:**
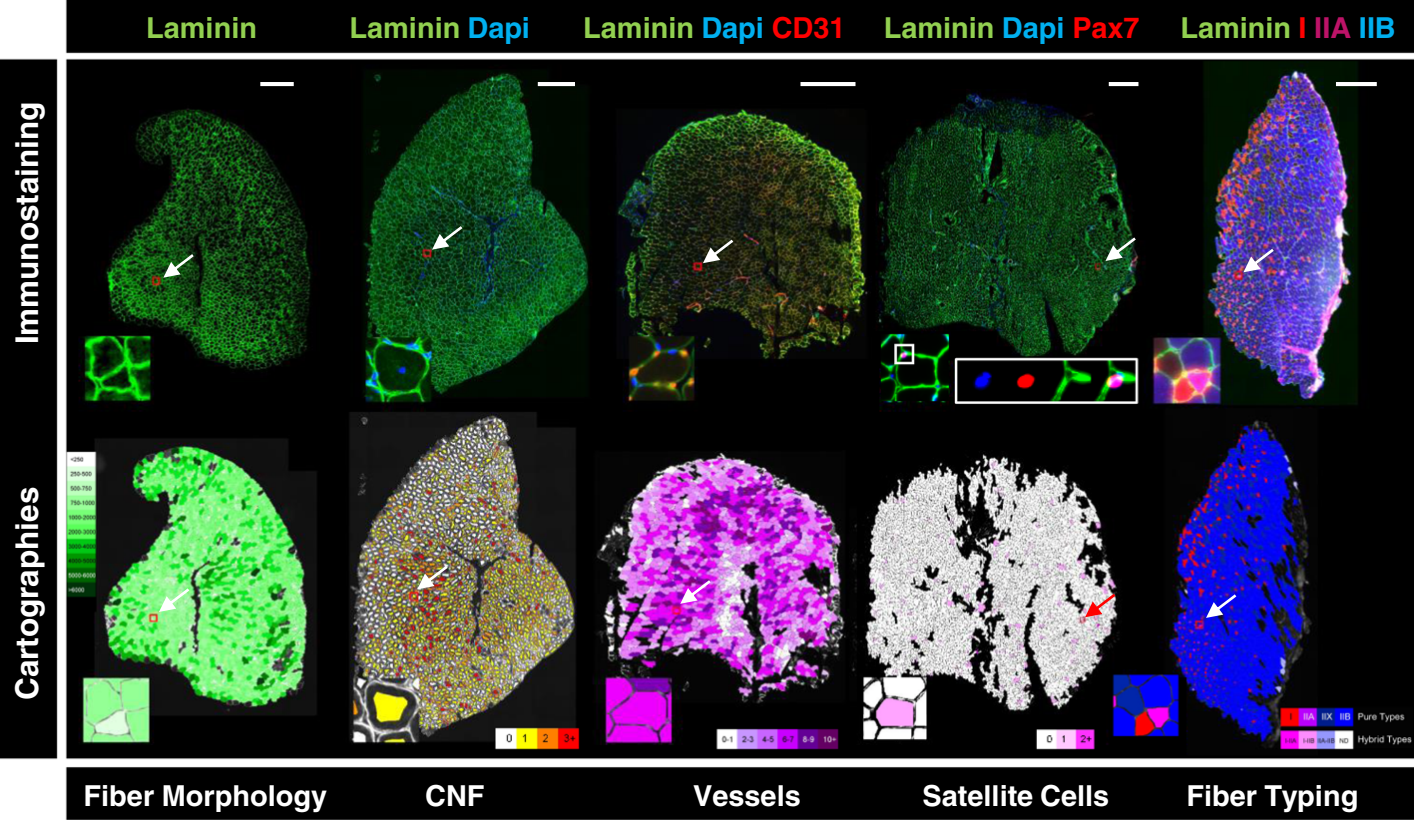
Scoring of skeletal muscle phenotypes. The upper panel represents entire skeletal muscle sections obtained from immunofluorescent staining with different antibodies (laminin, Pax7, CD31, myosin I, myosin IIA, myosin IIB) and Dapi. SB equals 500 μm (20×, AxioscanZ1). The lower panel represents cartographies made by MuscleJ of the respective stainings. Arrows indicate selections of enlarged fibers in boxes. For satellite cell, each channel is represented separately. The first cartography, colored with a green scale represents the cross-section area (CSA) of skeletal muscle fibers. The number of centronuclei, vessels per fiber (CD31^+^), satellite cells (Pax7+) per fiber and the fiber typing are represented by red, pink, violet, and purple-blue scales respectively. Color scales can directly be incorporated in the saved cartographies

To visually represent MuscleJ’s analysis, various in situ cartographies were implemented in the tool (Fig. 3). The first is CSA of fibers, where a green scale represents the distribution of different fiber CSA, from the smallest (in light green) to the largest (in dark green). The second corresponds to the number of centronuclei per fiber, with a white to red scale in which fibers without centronuclei are represented in white and the fibers with more than three centronuclei are represented in red. Cells with one and two centronuclei are represented with yellow and orange respectively. These cartographies can be used to localize muscle injury (Additional file 1: Figure S3), and cross-correlation analysis between different cartographies can also be performed.

The number of fiber-associated vessels (CD31-positive) can also be quantified per fiber and represented on a dedicated cartography where a purple scale defines six predefined classes of fibers: those associated with 0 to 1, 2 to 3, 4 to 5, 6 to 7, 8 to 9, and more than 9 vessels per fiber, from light to dark purple, allowing a precise analysis of the distribution vessel numbers per fiber. The number of satellite cells can also be quantified from images of co-staining with laminin, DAPI, and the specific marker of satellite cells, Pax7. Results are given per fiber, and a pink scale cartography was implemented for this parameter in order to directly distinguish on skeletal muscle sections, the fibers which are not associated with satellite cells (in white), from those containing one (in light pink) or more than one satellite cells (in dark pink).

Finally, MuscleJ was also designed to quantify up to three intrafiber stainings. Here we demonstrate fiber typing as an example. In this case, four different fiber types (type I, type IIA, type IIB, and a deduced type IIX) can be analyzed from three different stainings with specific antibodies. Type IIX fibers correspond to unlabeled fibers. Fibers that are positive for two stainings are named “hybrid fibers” (I-IIA, I-IIB, IIA-IIB), while fibers having more than two stainings are considered as “not determined” (ND) in results. A cartography is also associated with this intrafiber quantification allowing to easily represent results of these quantifications. MuscleJ gives the signal intensity for each fiber in each channel in the table of results.

### Validation of MuscleJ compared to benchmark methods used by different experts (Fig. 4)

**Fig. 4:**
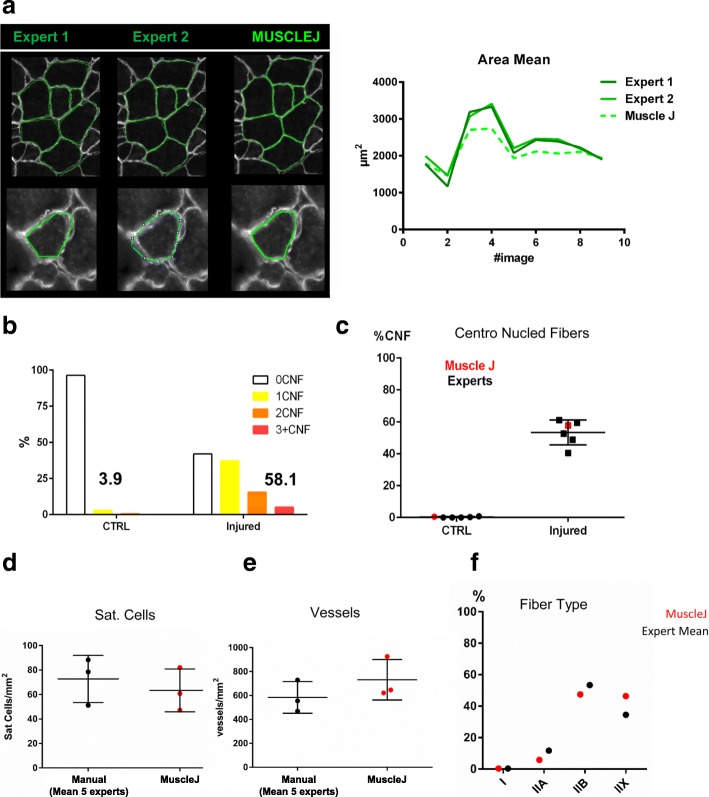
Method validation by feature. **a** The left panel represents the manual drawing of skeletal muscle fibers by two independent experts. The right panel is a graph representing the cross-section area (CSA) mean by expert compared to MuscleJ. **b** The percentage of fibers with no, one, two, or three and more centronuclei was quantified by MuscleJ on control skeletal muscle sections (left, CTRL, *n* = 4) and sections from injured skeletal muscle (right, *n* = 5). **c** Manual expertise by five independent experts compared to MuscleJ for the quantification of the percentage of centronucleated fibers. **d**, **e** Results obtained for manual quantification compared to MuscleJ for the number of satellite cells by mm^2^ (**d**), vessels by mm^2^ (**e**) and fiber type distribution. **f** For **d**, **e**, and **f**, each black dot represents the mean of manual quantification by five independent experts per image. Mann–Whitney test was used to compare manual and MuscleJ data for Sat. Cells and Vessels by mm^2^ (respectively, *p* = 0.70 and *p* = 0.40)

To validate the measured characteristics of fibers, two independent experts from two different laboratories manually surrounded each fiber labeled by laminin (Fig. 4a). Our results show similar relative results between experts and MuscleJ for the different images. One clear difference was an overestimation of the fiber CSA by the experts. This was likely due to the imprecision of the hand-drawing of fiber edges, notably for small fibers. MuscleJ has the advantage of outlining all fibers in a precise, reproducible manner.

The centronuclei detection functionality of MuscleJ was first validated by comparing the number of centronuclei from uninjured and regenerated muscle, 3 weeks after a notexin-induced injury. The results show that while only 3.9% of fibers have centronuclei in control conditions, MuscleJ detects the presence of 58.1% of fibers with centronuclei in injured muscles, the rest being non-regenerated fibers (Fig. 4b). Five independent experts from two different labs performed quantification of the same images and show similar results as those obtained with MuscleJ (Fig. 4c and Additional file 1: Figure S4A).

Other functionalities of MuscleJ (satellite cells, vessels, and intrafiber stainings) were also tested by these five experts (Fig. 4d–f). For satellite cells and vessels, the results were not different between the experts and MuscleJ, thus validating MuscleJ for analysis of such parameters. For the detection of intracellular staining, the results obtained by the experts were also consistent with those obtained using MuscleJ for type I and type IIA fibers. However, a Chi-square test indicates significantly different results for the distribution of type IIB and type IIX fibers (Fig. 4f), highlighting the difficulty of manually tracking such labelling (Additional file 1: Figure S4B-C). Consequently, comparison between different conditions with MuscleJ is more reliable than manual quantifications.

For all of these experiments, each expert recorded the time spent on each image. Upon comparison to the time required for image analysis by MuscleJ, we found that automated quantification was 10 to 30 times faster than manual quantification (Additional file 1: Figure S4D).

## Discussion

Histological characterization is of central interest in most studies of fundamental and clinical aspects of skeletal muscle pathophysiology. For these analyses, a variety of different tools have been developed using different software, but to date, there is no single tool capable of automatically analyzing all parameters simultaneously. As a result, each laboratory often develops its own strategy to quantify parameters of interest. Manual quantifications, mainly performed with Fiji, are often described in scientific articles. However, manual approaches are subject to technical and observer bias, questioning the reproducibility of results obtained by different laboratories and different image acquisition systems. In this study, we propose a high-content analysis tool developed on the free, open-source Fiji software, which we named MuscleJ. It allows quantification of many important skeletal muscle parameters, based on immunofluorescent staining of skeletal muscle sections and can be applied to all high-quality images obtained from a variety of microscopes.

As other available software, e.g., SMASH and Myovision, MuscleJ allows to analyze fiber morphology by measuring the number of skeletal muscle fibers, their CSA, and minimum and maximum Feret diameter after fiber segmentation based on laminin staining (Additional file 1: Table S3). In addition to laminin, up to three different intrafiber stainings can be quantified. Here, we highlight fiber typing based on myosin type I, type IIA and type IIB immunostainings. However, the use of other antibodies against specific proteins located within the fiber can also be analyzed in terms of number of positive fibers and intensity of the signal.

Another functionality of MuscleJ, also done by SMASH, is the identification of myonuclei localized in a central position within the fiber. The number of centronuclei per fiber can be now quantified by MuscleJ. Aberrant positioning of nuclei in the fiber is a feature shared by many muscle disorders [12–14]. This is also an important parameter for characterization of regenerating fibers as it is a readout of regeneration efficiency. Running MuscleJ on muscle sections co-labeled with laminin and DAPI returns the number of regenerated fibers and the number of nuclei in each fiber. Importantly, it can also report their respective CSA, and the combination of these different parameters is sufficient to show potential defects in the muscle regeneration process.

In addition, our tool presents unique functionality not currently available. We have developed the automatic detection of Pax7-positive muscle stem cells localized under the basal lamina of fibers, as well as CD31-positive endothelial cells of vessels in skeletal muscle. Both parameters are often quantified in muscle studies [15–17]. Analysis of CD31-positive cells, also performed by SMASH, offers here the possibility for combined analysis of satellite cells or intrafiber staining.

Combined analysis performed by MuscleJ is an original feature of this tool that is based on the possibility to track each individual fiber which is automatically numbered and saved in the ROI folder. Therefore, it is possible to establish correlations between the morphology, number of associated satellite cells and vessels, and number of centronuclei or intrafiber signals by fibers, depending on the antibodies used (see the “Possible analysis combinations” in the Additional file 1: Tutorial). In addition, the capacity of MuscleJ to quickly analyze all these parameters makes it possible to increase the amount of muscle sections to be analyzed and consequently improve statistical power.

Another original feature of MuscleJ is the optional in situ cartographies. Five different cartographies have been designed. The first one represents the number of centronuclei per fiber by a color code from white to red, where white fibers are normal fibers and red fibers correspond to those containing more than three nuclei in a central position. There are many advantages of this option, including the capability to directly visualize the extent of an injury induced by myotoxin injection or comparison of the degenerative state of different skeletal muscles (Additional file 1: Figure S2). The second cartography depicts the CSA of muscle fibers by a gradual color code ranging from pale to dark green. This tool offers a number of perspectives in studies focusing on skeletal muscle mass regulation where atrophy and hypertrophy are examined as an endpoint. The spatial information brought by this option can be used, for example, to determine the effect of local intramuscular injection of different treatments on fiber CSA. The three other cartographies present the number of satellite cells or vessels associated to fibers and the fiber type distribution. These cartographies can be optionally included in each analysis, depending on the box checked in MuscleJ when it is launched. The user should be aware that including additional analyses can greatly increase the running time of the program.

The high-content tool we developed is based on the analysis of different immunofluorescent stainings. Many studies, notably in the clinical diagnostic field, also use immunohistochemical staining to assess specific parameters, such as fibrosis by Sirius Red, metabolism by COX and SDH staining, glycogen content by PAS staining, and lipid content by Oil RedO. In the future, we plan to develop MuscleJ with new functions in order to detect and quantify these parameters. In addition, we will also add the possibility to analyze skeletal muscle of other species (human, horse, pig…) as our current algorithm has only been validated in mice. We plan to also develop the capacity of MuscleJ to align serial skeletal muscle sections labeled by immunofluorescent and immuno-histochemistry for specific markers. Finally, a plugin will be developed to further improve the interactive user interface and will be deposited on an appropriate open source web site for scientific community.

## Conclusions

MuscleJ has been designed to allow characterization of many parameters currently analyzed in fundamental and clinical studies in the skeletal muscle field. We have developed this tool on the free publicly accessible software Fiji, in order to offer the capacity to the muscle community to use it freely on high quality images obtained from a range of different microscopes. Using MuscleJ significantly reduces the time of analysis and renders possible the comparison of experiments performed at different times and in different laboratories around the world by providing highly reproducible analyses. The easy-to-use interface is highly intuitive and facilitates its usage. We expect that MuscleJ will become the tool of reference for all skeletal muscle histological analysis in the future.

## Additional file

Additional file 1: Supplementary tables, figures and tutorial. (PDF 2355 kb)

## Abbreviations

ATP: Adenosine triphosphate
CNF: Centronucleated fibers
COX: Cytochrome oxidase
CSA: Cross-section area
MDX: Mouse model of Duchenne muscular dystrophy
MRF: Myogenic regulatory factor
Nb: Number
ND: Not determined
PAS: Periodic acid Schiff
Pax7: Paired box protein 7
ROI: Region of interest
ROI^CNF^: Region of interest of centronucleated fibers
ROI^F^: Region of interest of fibers
ROI^SC^: Region of interest of satellite cells
ROI^V^: Region of interest of vessels
Sat Cell: Satellite cells
SMASH: Semi-automatic muscle analysis using segmentation of histology
TIF: Tagged image format
