# Supplementary material for: MuscleJ: a high-content analysis method to study skeletal muscle with a new Fiji tool
Source: Skelet Muscle. 2018 Aug 6;8:25. doi: 10.1186/s13395-018-0171-0 (PMC6091189; doi:10.1186/s13395-018-0171-0)

Supplementary information

MuscleJ: A high content analysis method to study skeletal muscle with a new Fiji tool

Alicia Mayeuf-Louchart*[1], David Hardy[2], Quentin Thorel[1], Pascal Roux[3], Lorna Gueniot[2], David Briand[2], Aurélien Mazeraud[2], Adrien Bouglé[2], Spencer L. Shorte[3], Bart Staels[1], Fabrice Chrétien[2], Hélène Duez[1], Anne Danckaert[*2,3]

1/ Supplementary Tables

2/ Supplementary Figures

3/ Tutorial

1/ Supplementary Tables

Supplementary Table1: Acquisition settings of microscope used for method validation on the same muscle section slide

		**Apotome**	**Confocal**	**Confocal Spinning Disk**
Analysis		Centro-nucleated fibers		
Image Dimensions	Z-Stack	4 Slices (6µm)	No	Yes
	Channels	2	3	2
	Scaling (per pixel)	0.258µmx0.258µmx2µm	0.38µmx0.38µm	
	Image Size (Scaled)	3.9mmx5.34mm	354.25µmx354.25µm	-
	Bit Depth	12 Bit	8Bit	
Acquisition Information	Microscope	Axio Observer Z1	LSM 700 AxioObserver	Cell Voyager 1000 (CV1000)
	Objective	25x	40x	40x
Channel1	Reflector	**DAPI**	**DAPI**	**DAPI**
	Excitation Wavelength	353	404	404
	Emission Wavelength	465	444	444
Channel2	Reflector	**FITC**	**Cy3**	**FITC**
	Excitation Wavelength	495	561	488
	Emission Wavelength	519	575	517
Channel3	Reflector		**FITC**	
	Excitation Wavelength		488	
	Emission Wavelength		517	

Supplementary Table2: Main FiJi functions called in the MuscleJ macro

Function Name	Parameters
**Pretreatment**	
"Gaussian Blur..."	Sigma=2 or 10
"Enhance Contrast"	Saturated parameter from 0.1 to 1.00 depending of image quality
"Subtract Background..."	Rolling=50
“setAutoThreshold”	Otsu (entire section) or Li (crop) threshold
**Morphology**	
"Find Maxima..."	noise= intensity mean given by histogram
"Analyze Particles..."	For Fiber : size=100-7000 circularity=0.45-1.00

Supplementary Table 3: Comparison of functionalities of SMASH, MyoVision and MuscleJ

	SMASH	MyoVISION	MuscleJ
Acessibility	MatLab (not free)	Free registration on website	Free/Fiji
Automatisation	Semi- automatic	No intervention of the user	No intervention of the user
File types	Bmp, Jpg, Png, Tiff 8 bit	Not indicated	Original files (CZI, LSM…), Tiff 16 bit
Automatic quality control of images	No	No	Yes
Fiber morphology	Yes	Yes	Yes
Min and Max Feret dia	Yes	Yes	Yes
Detection of satellite cells	No	No	Yes
Intra-fiber straining	Yes	Yes	Yes
Detection of myonuclei	Yes	Yes	Yes
Detection of centronuclei	Yes	No	Yes
Quantification of centronuclei	No	No	Yes
Detection of vessels	Yes	No	Yes
Correlation of vessels and satellite cells	No	No	Yes
Cartographies	No	No	Yes
Automatic backup of results	Not indicated	Not indicated	Yes
Concordance expert/software on control muscle sections	86% (for fiber typing and fiber CSA, results were not compared by experts but with publish data)	98,2%	99.7%
Concordance expert/software on Regenerating muscle sections	Not indicated	Not indicated	79%

2/ Supplementary Figures

Supplementary Legends

Supplementary Figure 1: (A) Quick quality check by automatic artefact tracking Major artefacts (red panel) and ambiguous analysis (orange panel) **(B)** Fiber artefact detection corresponding to a low circularity or a high area.

Supplementary Figure 2: (A) Cartographies representing centronucleated fibers (upper panels) and fiber cross section area (lower panels) of control and Mdx skeletal muscles. **(B)** Distribution of fiber frequency with same CSA range in control and Mdx mice.

Supplementary Figure 3: Cartographies representing centronucleated fibers (upper panels) and fiber cross section area (lower panels) of control and injured. Cartographies of centronucleated fibers indicate that muscle injury is mainly limited to the right part of the muscle section, compared to Mdx muscles where centro-nucleated fibers are homogeneously distributed along the section. Comparison of the two cartographies shows that centronucleated fibers (yellow, orange, red) are smaller than non-centronucleated fibers (white).

Supplementary Figure 4: (A) Concordance Matrix of results (expressed in percentage) on injured skeletal muscle sections and control sections without injury. Concordance accuracy (in bold) are respectively 99.7% and 79%. The False Negative (in dark gray) are respectively 0.2% and 17%, respectively. False Positive (in light grey) are respectively 0.1% and 3%, respectively. **(B)** Representation of the number of Type I, IIA, IIB, IIX fibers quantified by 5 independent experts on 2 different images. Error bars represent the variability of replicate measures per expert. **(C)** Fiber type distribution comparison between experts and MuscleJ for two different images. Hybrid fibers are not represented in the graphs of panels B and C. **(D)** Mean of related time consuming by fiber by experts and MuscleJ, for the quantification of different parameters.

Supplementary Figure 1

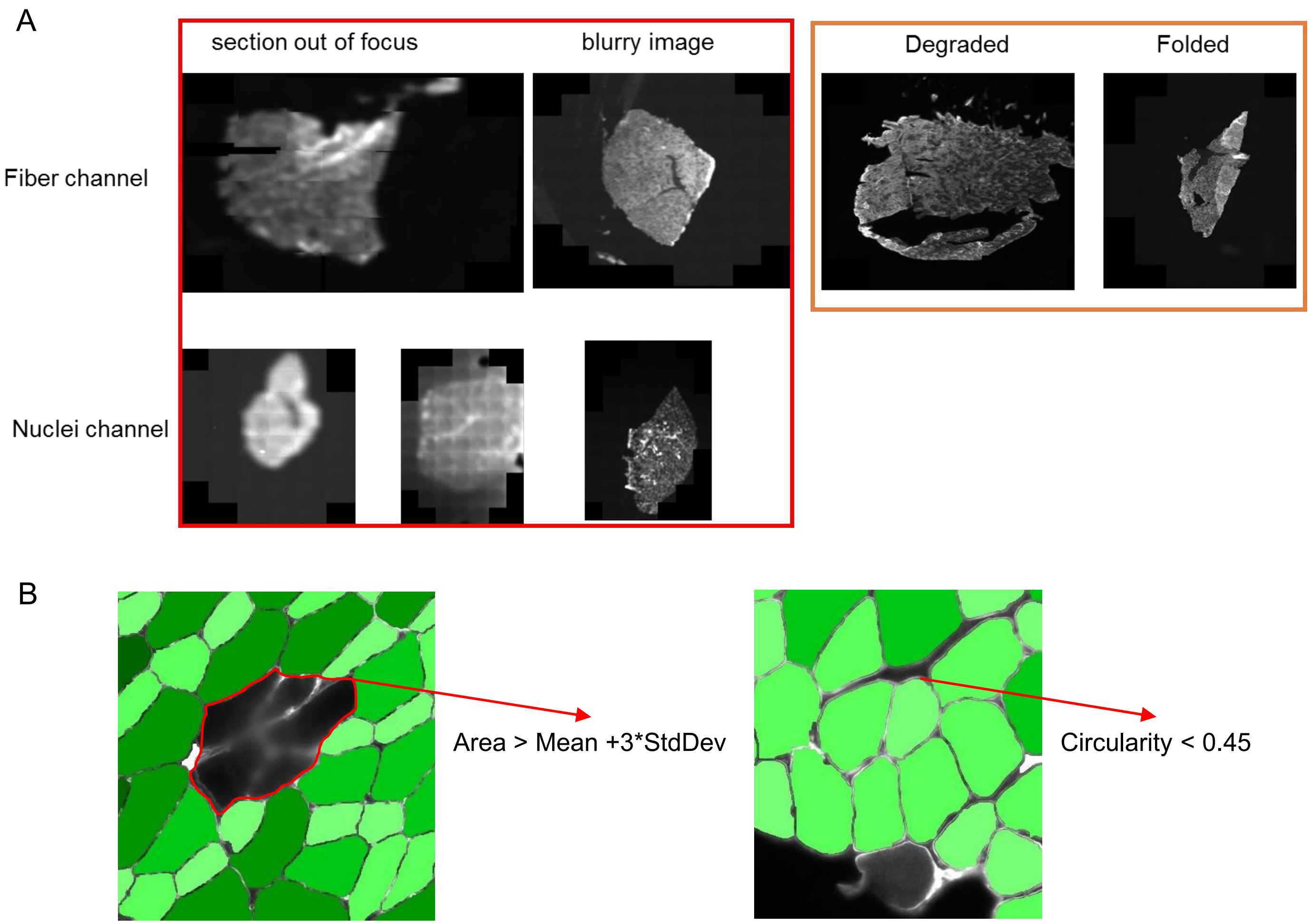

Supplementary Figure 2

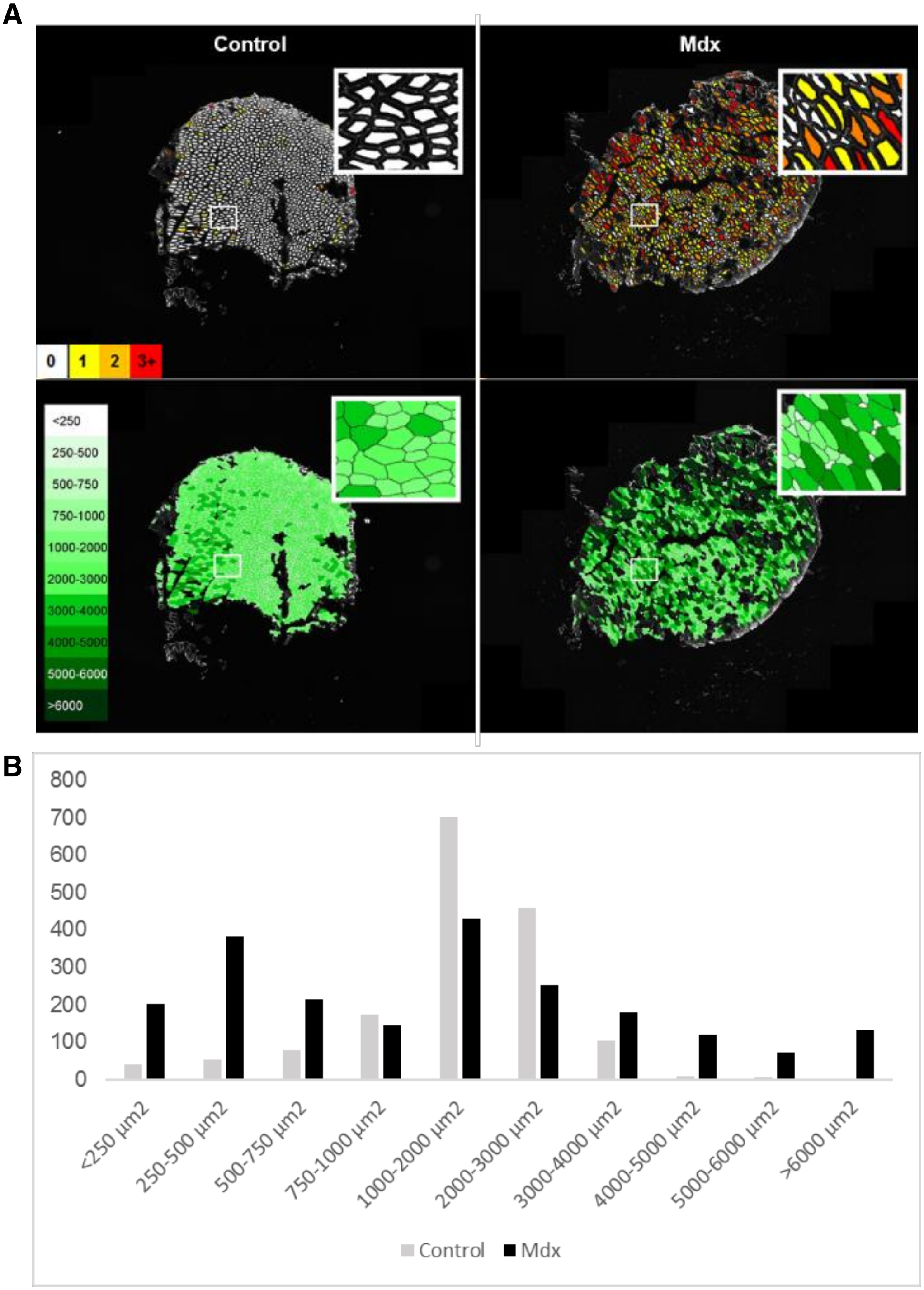

Supplementary Figure 3

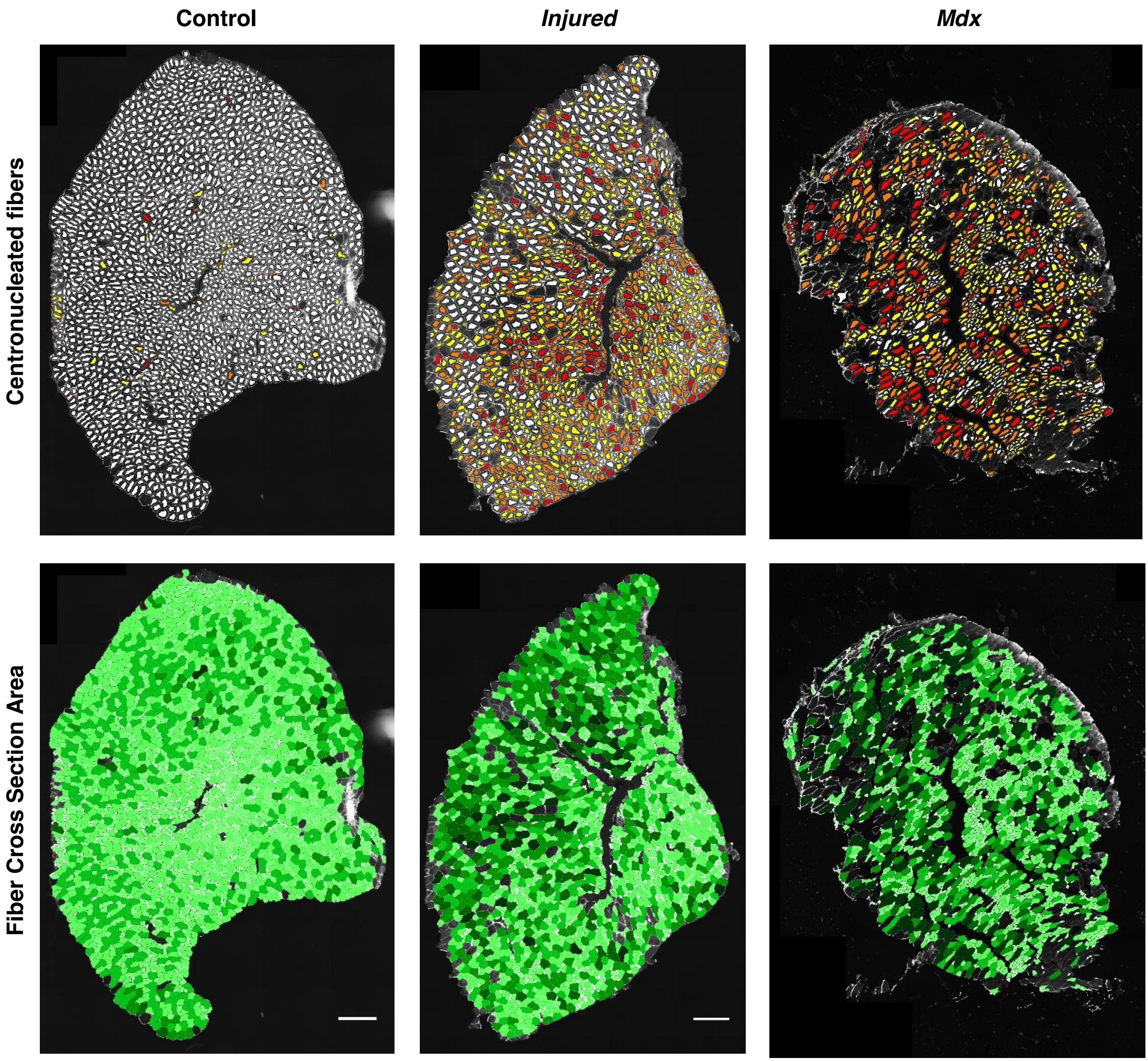

Supplementary Figure 4

A

Controls

E \ M	0	1	2	3+	
0	99,7%	0,2%	0,1%	0,0%	0,2%
1	0,1%	0,0%	0,0%	0,0%	
2	0,0%	0,0%	0,0%	0,0%	
3+	0,0%	0,0%	0,0%	0,0%	
	0,1%				**99,7%**

Injured

E \ M	0	1	2	3+	
0	39%	13%	4%	0%	17%
1	2%	20%	4%	1%	
2	1%	3%	7%	1%	
3+	0%	0%	2%	2%	
	3%				**79%**

B

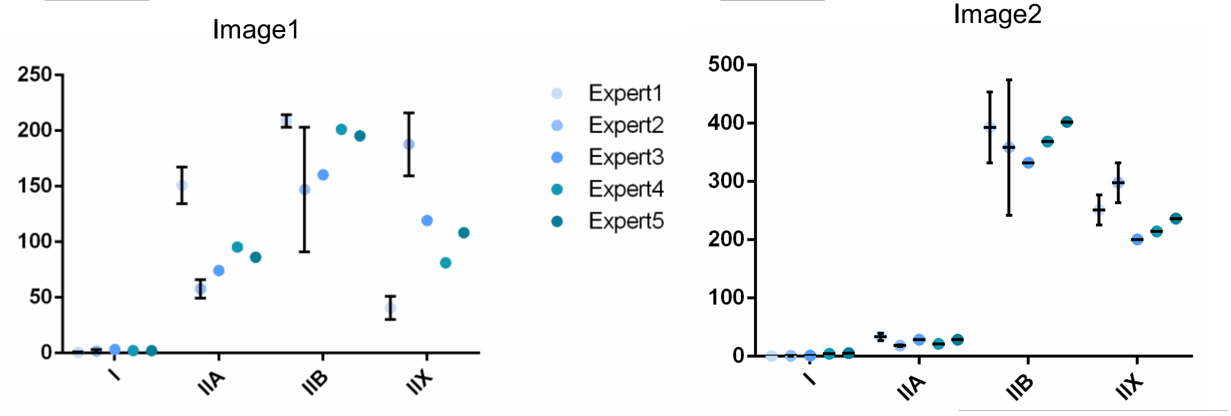

C

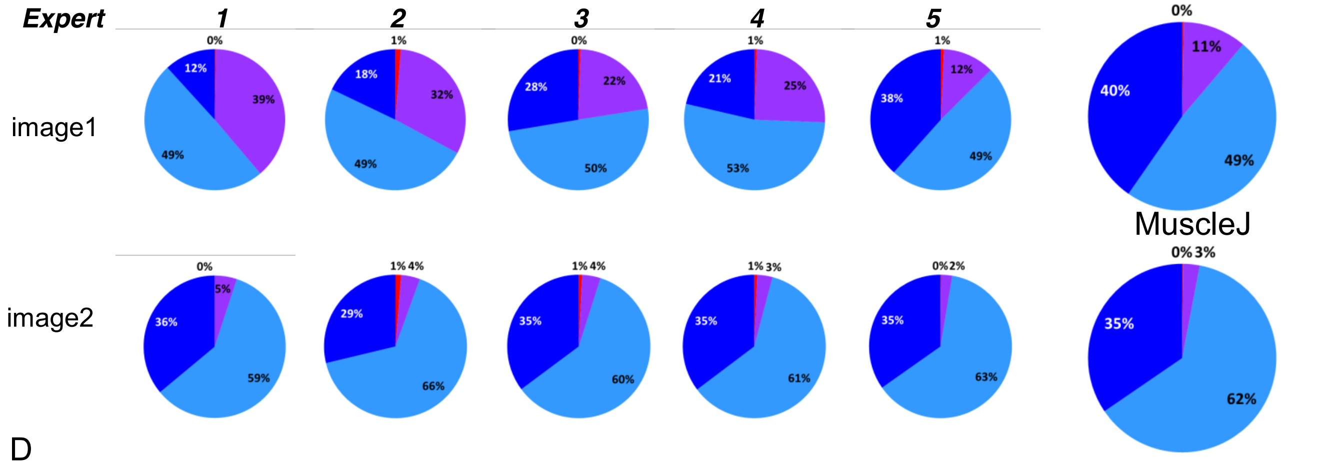

D

Features	Nb expertised Fibers	Expert Time (s)	MUSCLEJ (s)
CNF	919	2.6	0.26
Vessels	729	1.9	0.06
Type	1566	0.9	0.04
Sat. Cells	1188	0.6	0.04

3/ Tutorial

The tutorial will be posted on the website where MuscleJ will be deposited. Both will be updated regularly depending on new features or major bug corrections.

MuscleJ Tutorial

MuscleJ: A high content analysis method to study skeletal muscles.

The MuscleJ macro is a compilation of tools allowing for the analysis of fiber phenotypes.

Corresponding Authors

Anne Danckaert – anne.danckaert at pasteur.fr - Institut Pasteur, Paris, France.

Alicia Mayeuf-Louchart - alicia.mayeuf-louchart at pasteur-lille.fr - Institut Pasteur de Lille, U1011 - EGID, Lille, France.

Features

Fiber morphology – Centro Nuclei Fiber detection (CNF) – Vessel detection – Satellite Cell detection (Sat) – Fiber typing

Contents

I. Installation of MuscleJ in the FiJi environment
II. Description
   1. The user provides the requested information by dialog box
   2. Setting the directory path for the Input and the Output data
III. Possible analysis combinations
IV. Recommendations / Limitations
V. Examples

I. Installation of MuscleJ in the FiJi environment

The automated image analysis workflow was implemented in the FiJi (NIH, Bethesda, MD, USA) environment as a macro and will be upgrade to a plugin with further functionalities.

Installation

- Download FiJi from https://imagej.net/Fiji/Downloads and following the installation instructions. Or update your FiJi version to meet the minimum version requirements.
- The minimum version requirements are:
  Fiji version from 1.51e, tested on 1.52a
  Java version: Java 1.8.0-66 (64 bits)
  Used Plugins: Bio-Formats Plugins for Fiji
  (from release 5.5.3, tested on 5.8.2)

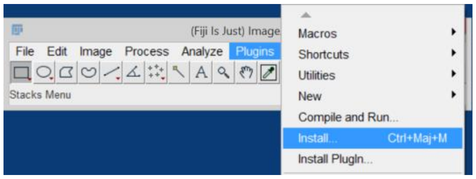

- Installation of Muscle J
  - Download the MuscleJ_V1_00.ijm file onto your computer.
  - From *Plugins➔Install…* menu, open the MuscleJ macro and save it into the FiJi 'Plugins/Macros' folder.
  - Restart FiJi to complete the macro installation.
  - MuscleJ will appear in the *Plugins➔Macros* menu.

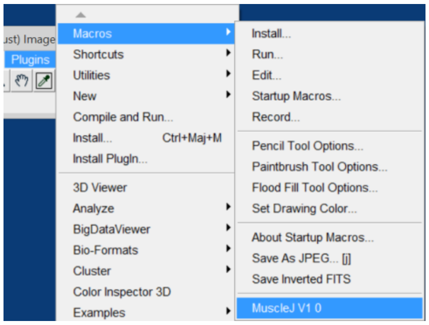

I. Description: MUSCLEJ Fiber Phenotype Dialog Boxes

The first step : The user provides the requested information

Data Acquisition

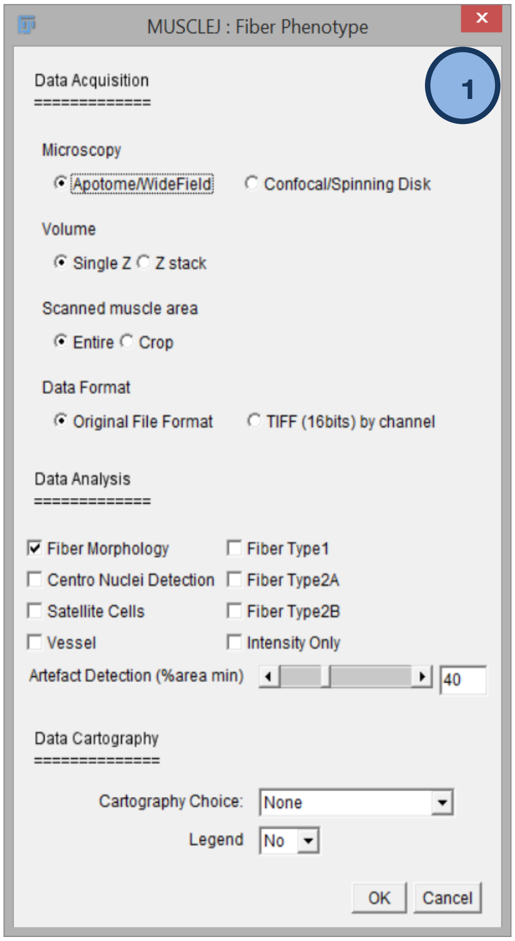

- *Microscopy* selection
  Select the type of microscopy used when generating the data. Based on the selection a specific pretreatment will be performed.
- *Volume* choice
  The *Z stack* option will apply a maximum projection along z axis.
- *Scanned muscle area* choice
  Entire: the whole section or a large part of the section (min. 50%) will be been scanned.
  Crop: if only a small area is to be scanned the analysis will be less efficient despite a series of additional filters that track the maximum intensity of the fiber contours in Laminin channel.
- *Data Format*
  The *Original File Format* is for files readable by the *Bioformat Importer* Plugin such as czi, lsm, lif, etc. formats. Additionally, tiff files containing all channels (a stack file from FiJi or ImageJ) are readable.
  The *TIFF (16bits) by channel* format is for cases when your images have been exported from your acquisition system by channel.

Data Analysis

- *Analysis* check boxes: multiple options – you can select the series of analysis to be performed. However, the *Fiber Morphology* analysis is mandatory whenever you start a new batch run.
- *Artefact Detection* option: sets the minimum threshold for the fiber area detection. This is used for the initial quality check of the fiber shape signal (Laminin).
  Example: %area min=xx, if less than xx% of the total area of the section does not contain segmentable fibers, the section will be automatically placed into the "Artefact" directory and the analysis will not be performed for this section.

Data Cartography

- *Cartography*
  Automatically backs up the *in situ* cartography according to the analysis performed (single choice): "*Fiber Area Classes*", "*Centro Nuclei Classes*", "*Sat Cell*", "*Fiber Types*" or "*Vessels*". Note: If “*Fiber Morphology*” analysis has been performed during a previous run, the corresponding cartography ("Fiber Area Classes") can be chosen independently from the current analysis.
- *Legend*
  If chosen, for the Centro Nuclei Fibers, Sat Cells, Types, and Vessels a legend will be automatically drawn onto the cartography *in situ* on the bottom left. For the distribution by fiber surface the legend is put in a column at the top left of the cartography with the range of surface distribution displayed in μm^2.

Click on the OK button to continue.

Channel Information

To identify the channel order, please open the first image from the input folder with the *Bioformat Importer*.

Depending on the analysis requested by the user, the order of channels will be need to be set.

In the right dialog box, the analysis of fiber typing has been demanded. You must enter the corresponding fiber channel number to the track of the fiber shape, before the analysis of intensity by fiber typing channel can be performed.

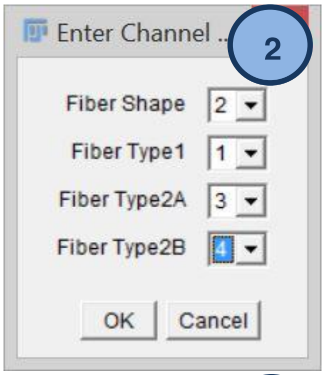

For the same analysis request, if the *tiff format by channel* option has been checked the information about the number of channels, the x pixel size in microns and the y pixel size in microns must be entered before the analysis can be performed.

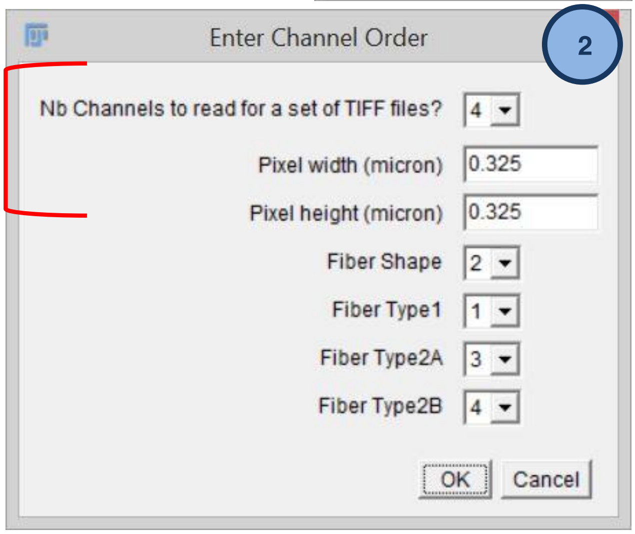

CClick on the OK button to continue.

Note: all fields in the two previous dialog boxes are mandatory

The second step: Setting the directory path for the Input and the Output data

- Select the Image File Folder by Batch run
- Select an empty folder to save the ROIs, the Results by file and the Cartographies During the first run, a series of directories will be automatically created in the root of the selected folder:

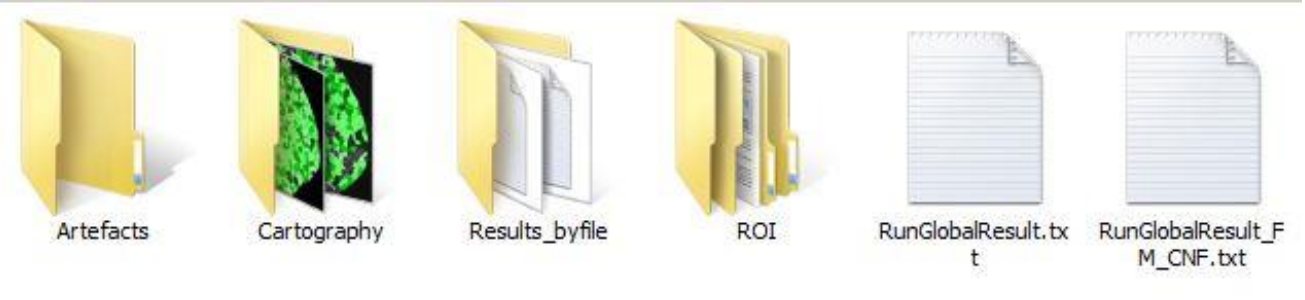

II. Possible analysis combinations

One channel is dedicated to the detection of the shape of the fiber (Laminin).

+ 1 channel	+ 2 channels	+ 3 channels
• CNF (Dapi)<br>• Vessels (CD31)<br>• Intrafiber staining (Fiber type or other intra-staining)<br>• Intensity per channel	• Sat Cells (Dapi+Pax7)<br>• CNF (Dapi) + Vessels (CD31)<br>• CNF (Dapi) + Sat Cells (Pax7)<br>• CNF (Dapi) + one intrafiber staining<br>• Two intrafiber staining	• CNF (Dapi) + Sat Cells (Pax7)+ Vessels (CD31)<br>• Three intrafiber staining (3 Fiber types or other intra-staining)<br>• CNF (Dapi) + Vessels (CD31) + One intrafiber staining<br>• CNF (Dapi) + Sat Cells (Pax7)+ one intrafiber staining<br>• CNF (Dapi) + two intrafiber staining.

III. Recommendations / Limitations

Images obtained from any microscope magnification (10x, 20x, 25x, 40x, 63x) can be used. The limitations are relative to the quality of image staining and a minimum of 1.5x1.5mm scanned surface area.

For an optimal utilization of MUSCLEJ we recommend using the tool under the following conditions:

When gather the data use an Apotome / Widefield microscope when imagining a single Z slice or use a confocal/Spinning Disk microscope when imaging a Z-stack. When analyzing a whole section (set the minimum detection threshold of 40 % to reject section artefacts).

Use the Original File Format to keep the metadata associated.

Images folder: all images of the batch folder should have the same parameters (number of channels, channel orders, stainings and formats). The input and output folders have to be named without using spaces and symbols.

File format that are not supported are: Jpeg, Png, Tiff-8bits, a time series.

For the option « *Tiff 16 bits by channel»:* Each file must contain one channel and have the following nomenclature **FileName1_C#** where # is the number of channel (1,2,3 or 4). Do not use spaces and symbols when naming the files.

Note: All the files have to be gather into the image folder

IV. Examples

Data set description

Input Folder Name	Channel Order	Acquisition	Raw Data	Output Folder
• CNF<br>• FiberMorphology	Dapi(1)<br>Laminin(2)	Widefield 20X	Yes	• ResultsCNF<br>• ResultsFMorpho
Fiber Type	Laminin(1)<br>MyhI(2)<br>IIB(3) IIA(4)	Widefield 20X	Yes	ResultsTypes
SatCells	Dapi(1)<br>Laminin(2)<br>Pax7(3)	Widefield 20X	No	ResultsSatCells
Vessels	Dapi(1)<br>CD31(2)<br>Laminin(3)	Widefield 20X	Yes	ResultsVessels

All the data corresponding to the described analysis can be downloaded from github site via a request to the corresponding authors.

Single analysis

Analysis	Options to check	Results by batch run	Results by fiber
Fiber Morphology	• Apotome/Widefield<br>• Single Z<br>• Whole section<br>• Original file format<br>• Quality Check: 40%<br>• Cartography<br>• No legend	***GlobalResults_FM.txt***<br>• Whole section area (μm^2)<br>• Numbers of segmented fibers<br>• Fiber Area mean (μm^2)<br>• Distribution of the fiber areas.	• Area<br>• Minimum and maximum Ferret<br>• 2D Localisation (GC)
+CNF	• Apotome/Widefield<br>• Single Z<br>• Crop<br>• Original file format<br>• Quality Check: 40%<br>• No Cartography	***GlobalResults_FM_CNF.txt***<br>• Total Centronucleated fibers	• Area<br>• Minimum and maximum Ferret<br>• Numbers of centronuclei<br>• Numbers of perinuclei
+Sat Cells	• Apotome/Widefield<br>• Single Z<br>• Crop<br>• Original file format<br>• Quality Check: 40%<br>• Cartography<br>• No Legend	***GlobalResults_FM_SC.txt***<br>• Numbers of satellite cells (Pax7$^+$ cells)<br>• Total of satellite cells associated fibers	• Area<br>• Minimum and maximum Ferret<br>• Numbers of satellite cells associated to the fiber
+Vessels	• Apotome/Widefield<br>• Single Z<br>• Crop<br>• Original file format<br>• Quality Check: 40%<br>• Cartography<br>• No Legend	***GlobalResults_FM_V.txt***<br>• Total of vessels<br>• Total of vessels associated fibers<br>• Vascularisation Surface(%)<br>• Number of vessels by mm^2	• Area<br>• Minimum and maximum Ferret<br>• Numbers of vessels associated to the fiber

Multiple analysis

Analysis	Option check	Results by batch run	Results by fiber
FM+ 3 Fiber Types	• Apotome/Widefield<br>• Single Z<br>• Crop<br>• Original file format<br>• Quality Check: 40%<br>• Cartography<br>• Legend	***GlobalResults_FM_I_IIA_IIB.txt***	• Area<br>• Minimum and maximum Ferret<br>• 2D Localisation (GC)<br>• Fiber typing

FM+CNF+Vessels	• Apotome/Widefield<br>• Single Z<br>• Crop<br>• Original file format<br>• Quality Check: 40%<br>• Cartographies<br>• Legend	***GlobalResults_FM_CNF_V.txt***<br>• Tot Centro Nuclei<br>• Tot Vessels<br>• Fibers with Vessel<br>• Vascularisation surface(%)<br>• Vessels by mm^2	• Area<br>• Minimum and maximum Ferret<br>• 2D Localisation (GC)<br>• Numbers of centronuclei<br>• Numbers of perinuclei<br>• Numbers of vessels associated to the fiber

Example of cartographies corresponding to multiple analysis

FM+CNF+Vessels

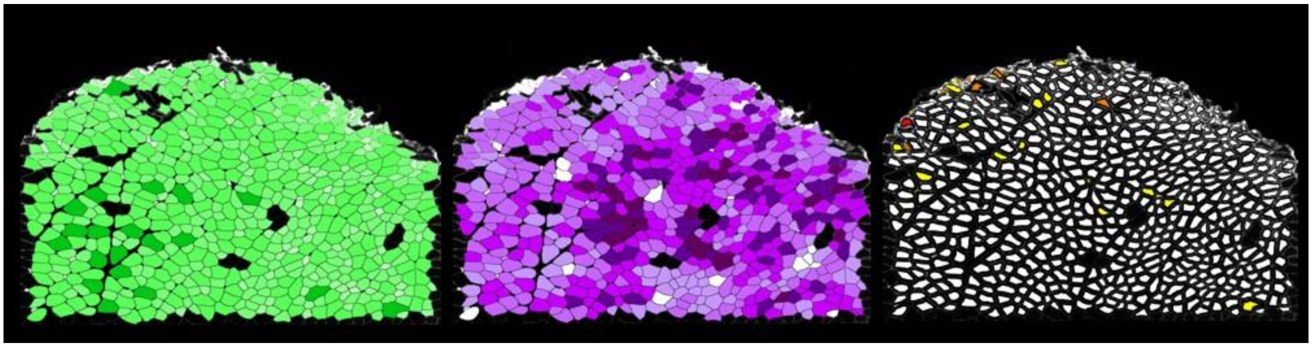

Supplement: Supplementary file 1 — Supplementary tables, figures and tutorial. (PDF 2355 kb) [file 13395_2018_171_MOESM1_ESM.pdf]
